# Circadian Remodeling of Neuronal Circuits Involved in Rhythmic Behavior

**DOI:** 10.1371/journal.pbio.0060069

**Published:** 2008-03-25

**Authors:** María Paz Fernández, Jimena Berni, María Fernanda Ceriani

**Affiliations:** Laboratorio de Genética del Comportamiento, Instituto Leloir, Instituto de Investigaciones Bioquímicas-Buenos Aires–Consejo Nacional de Investigaciones Científicas y Técnicas (IIBBA-CONICET), Buenos Aires, Argentina; University of Geneva, Switzerland

## Abstract

Clock output pathways are central to convey timing information from the circadian clock to a diversity of physiological systems, ranging from cell-autonomous processes to behavior. While the molecular mechanisms that generate and sustain rhythmicity at the cellular level are well understood, it is unclear how this information is further structured to control specific behavioral outputs. Rhythmic release of pigment dispersing factor (PDF) has been proposed to propagate the time of day information from core pacemaker cells to downstream targets underlying rhythmic locomotor activity. Indeed, such circadian changes in PDF intensity represent the only known mechanism through which the PDF circuit could communicate with its output. Here we describe a novel circadian phenomenon involving extensive remodeling in the axonal terminals of the PDF circuit, which display higher complexity during the day and significantly lower complexity at nighttime, both under daily cycles and constant conditions. In support to its circadian nature, cycling is lost in bona fide clockless mutants. We propose this clock-controlled structural plasticity as a candidate mechanism contributing to the transmission of the information downstream of pacemaker cells.

## Author Summary

Circadian systems evolved as a mechanism that allows organisms to adapt to the environmental changes in light and dark which occur as a consequence of the rotation of Earth. Because of its unique repertoire of genetic tools, *Drosophila* is a well established model for the study of the circadian clock. Although the biochemical components underlying the molecular oscillations have been characterized in detail, the mechanisms used by the clock neurons to convey information to the downstream pathways remain elusive. In the fruit fly, the small ventral lateral neurons (LNv) are capable of synchronizing other clock cells relying on a neuropeptide named pigment dispersing factor. In this work we introduce a novel mechanism as a possible candidate for contributing to the transmission of information downstream of the small LNvs, involving clock-controlled remodeling of their axonal morphology. By labeling the entire neuronal membrane and analyzing the complexity of the axonal arbor at different times we showed that there is a circadian variation in the complexity of the axonal arbor. This phenomenon was not observed in flies carrying null mutations in two canonical clock genes, underscoring the dependence of the circadian clock for the structural plasticity of its pacemaker neurons.

## Abstract 3

The circadian clock controls a wide array of biological phenomena ranging from basal transcription to overt behavior. Now, new evidence shows that the clock affects a striking remodeling of the circuit controlling rest-activity cycles in*Drosophila*.

## Introduction

Many organisms display daily rest–activity cycles, which are reminiscent of the sleep–wake cycles observed in humans. This rhythmic activity is sustained even in the absence of environmental light-dark cues, revealing its endogenous origin. Along the years, many of the components of the circadian clock responsible for generating and sustaining molecular rhythmicity have been identified and characterized in different model systems [1,2], but only recently a picture of how molecular rhythms operating at a single cell level are translated to overt rhythmic behavior is beginning to unfold in *Drosophila*.

Rhythmic locomotor behavior in flies depends on an intact molecular oscillator [3] operating within the circadian circuitry, which is comprised by three major neuronal clusters (reviewed in [4,5]). The small ventral lateral neurons (LNv) are at the top of the hierarchy because they command not only the pace of most of the remaining clusters [6], but also direct rhythmic behavior in the absence of any other brain oscillators [7] or by themselves in a clockless mutant [8]. The small LNvs send their projections towards the dorsal protocerebrum where some of the dorsal clusters are located [9,10], and release a neuropeptide known as pigment dispersing factor (PDF) in a circadian fashion [11]. Supporting its link to clock-controlled locomotor behavior, *pdf* levels are affected in arrhythmic bona fide clock mutants [11–13]. Moreover, PDF itself has been considered crucial in sustaining behavioral oscillations, after a thorough analysis of the effect of altering *pdf* levels [14,15] and its role in the synchronization between the different brain oscillators [6,16,17], some of which have recently been shown to express PDF receptor [18–20]. PDF levels in the dorsal protocerebrum change throughout the day likely not as the result of posttranslational peptide processing or transport per se but as a consequence of differential release [11]. However, recent data suggests that PDF cycling in dorsal protocerebrum is not necessary for the maintenance of rhythmic behavior in DD, since overexpression of a fusion protein between the rat atrial natriuretic factor and the green fluorescent protein (GFP) [21] collapses the oscillation in PDF levels (measured as signal intensity) while behavioral rhythmicity is largely unaffected [22].

In experiments involving PDF staining at different times during a daily cycle we noticed that the arborizations of the small LNv dorsal projections changed between early morning and early night to a higher degree than that anticipated simply by the variation in PDF intensity. To characterize in depth these daily changes in the PDF circuit, a membrane-bound fluorescent reporter was used to mark the entire structure. Here we demonstrate that the circuit underlying rhythmic behavior undergoes cyclic changes in the topology of its dorsal termini under synchronizing light-dark cycles and even in the absence of environmental cues, underscoring its connection with the endogenous biological clock. Moreover, this daily variation in circuit structure is abolished in arrhythmic clock mutants (such as *per^01^* and *tim^01^*), further supporting its circadian nature. We propose that circadian remodeling of the PDF circuit reveals an additional layer of clock control towards the consolidation of rhythmic rest-activity cycles.

## Results/Discussion

### Daily Changes in the Circuit Underlying Rhythmic Rest–Activity Cycles

**Figure 1:**
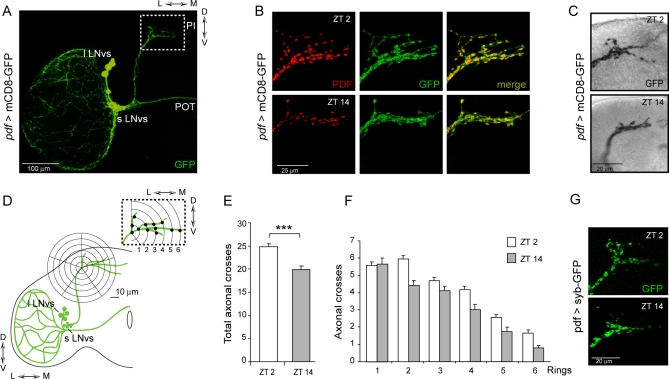
Daily Reorganization in the PDF Terminals at the Dorsal Protocerebrum (A–F) *pdf* >mCD8-GFP wild-type brains were dissected at ZT2 and ZT14, that is 2 h after lights ON and light OFF, respectively. Brains were stained with anti-GFP (green) and anti-PDF (red) antibodies. sLNvs and lLNvs stand for small and large lateral neurons ventral. (A) Low magnification view comprising a brain hemisphere. The region subject to analysis is shown in the inset. (B) Representative confocal images taken during the early day and early night, underscoring the striking reorganization taking place within this area. (C) HRP staining on *pdf* >mCD8-GFP brains to qualitative confirm under DIC the anatomical properties of the daytime and nighttime conformation as seen by confocal microscopy. (D) Schematic diagram depicting how the quantitation of the complexity of the PDF axonal arbor on confocal images was carried out. (E) The total number of intersections between the concentric rings and the axonal projections was significantly different in wild-type brains dissected during the early day and early night (*** represent *p* < 0.0001, non parametric Mann-Whitney test). Immunohistochemistry was performed at least three times, each timepoint represents a minimum of 40 brains; quantitation was performed blind. (F) The complexity of the axonal arbors is consistently lower in the nighttime conformation. (G) Expression of the presynaptic marker synaptobrevin-GFP (*pdf* >syb-GFP) shows a differential arrangement of synaptic vesicles at these two timepoints. Flies were dissected at ZT2 and ZT14.

Rhythmic locomotor behavior heavily relies on the concerted action of the small LNvs. These PDF-expressing neurons extend their projections dorsally towards the protocerebrum forming an axonal stem, which halfway through its trajectory shifts towards the center of the brain and opens up into a 2-D axonal arbor (Figures 1A–1C and Figure S1). Expression of synaptobrevin-GFP (a presynaptic marker) supports the notion that these arborizations are axonal projections (Figure 1G) [23].

PDF intensity levels cycle within the dorsal protocerebrum (Figure 1B, left panel) [11], but whether this cycling results in a modulation of its postsynaptic response has yet to be established; interestingly, dense-core vesicles reactive to a PDF-like peptide have been described in the dorsal protocerebrum of *Musca* and *Drosophila* [24], suggesting that their accumulation and release could be under clock control, as it appears to be the case for the dense-core vesicles found in the lamina [25]. While characterizing these PDF changes at the dorsal protocerebrum in greater detail we noticed that the PDF signal displayed a distinct configuration at different times in the day, which could not be simply attributed to the circadian control of PDF levels. We speculated that this observation could also result from plastic changes in the anatomical properties of the axons themselves. In fact, daily and circadian changes in morphological features of the axonal projections have been reported in other circuits, although none of them has a clear connection with a specific physiological output [25–28].

In an attempt to characterize in depth daily changes in the PDF circuit, a transgenic line expressing a highly stable, membrane-tethered version of the GFP (mCD8-GFP [29]) under the *pdf* specific promoter [14] was used. Thus, mCD8-GFP recreates the entire PDF circuit, that is, the small and large LNvs somatas as well as their axonal and dendritic projections. By using a stable reporter as opposed to the neuropeptide itself should ensure that any observed variations in circuit morphology could not be attributed to its expression, post-translational processing or transport. Transgenic *pdf-gal4;* UAS-mCD8-GFP flies (from now on referred to as *pdf* >mCD8-GFP) were synchronized to 12 h light: 12 h dark cycles to entrain their molecular oscillators. Young (0–3 d old) adult brains fixed at *zeitgeber time* (ZT) 2 and 14 (ZT2 and ZT14 indicate 2 h after lights ON and light OFF, respectively) were stained with anti-PDF and anti-GFP antibodies. These time-points were selected as they represent the peak and the trough of PDF intensity [11]. Figure 1B shows representative images of each condition in a wild-type background. Not only did the PDF-associated signal change during early morning and early night (compare ZT2 to ZT14, left panel) but also and most strikingly, the circuit itself, revealed through GFP staining, underwent these daily fluctuations at the level of the dorsal projections. In the early morning, it showed a higher degree of arborization, including both more numerous and likely higher order axonal processes. In contrast, at nighttime, the circuit adopted a closed conformation, where fewer branches could be distinguished. A magnified view of these projections stained with the avidin-biotin complex method is shown in Figure 1C.

The degree of axonal arborization was quantified by using an adaptation of Sholl's method to study the dendritic branching pattern [30]. As depicted in the cartoon in Figure 1D the number of intersections between the concentric rings and the projections were determined. Representative images illustrating how the intersections were visualized and quantitated are included in Figure S2A. A significant difference in the axonal morphology was observed (*p* < 0.0001, non parametric Mann-Whitney test), whereby at nighttime there was a decrease in the complexity of the axonal arbor of the PDF circuit (Figure 1E), represented by fewer intersections. Noteworthy, when presynaptic structures were visualized using a synaptobrevin-GFP fusion protein, a differential pattern of vesicle distribution was observed, suggesting the possibility that the activity is concomitantly changing (Figure 1G and unpublished data). One possible scenario to account for this observation is that synaptic contacts are remodeled as a function of time of day, or alternatively, that the PDF terminals contact different targets at different times. At present we cannot exclude either possibility.

### Plastic Changes in the Circuit Underlying Rhythmic Behavior Have an Endogenous Origin

**Figure 2:**
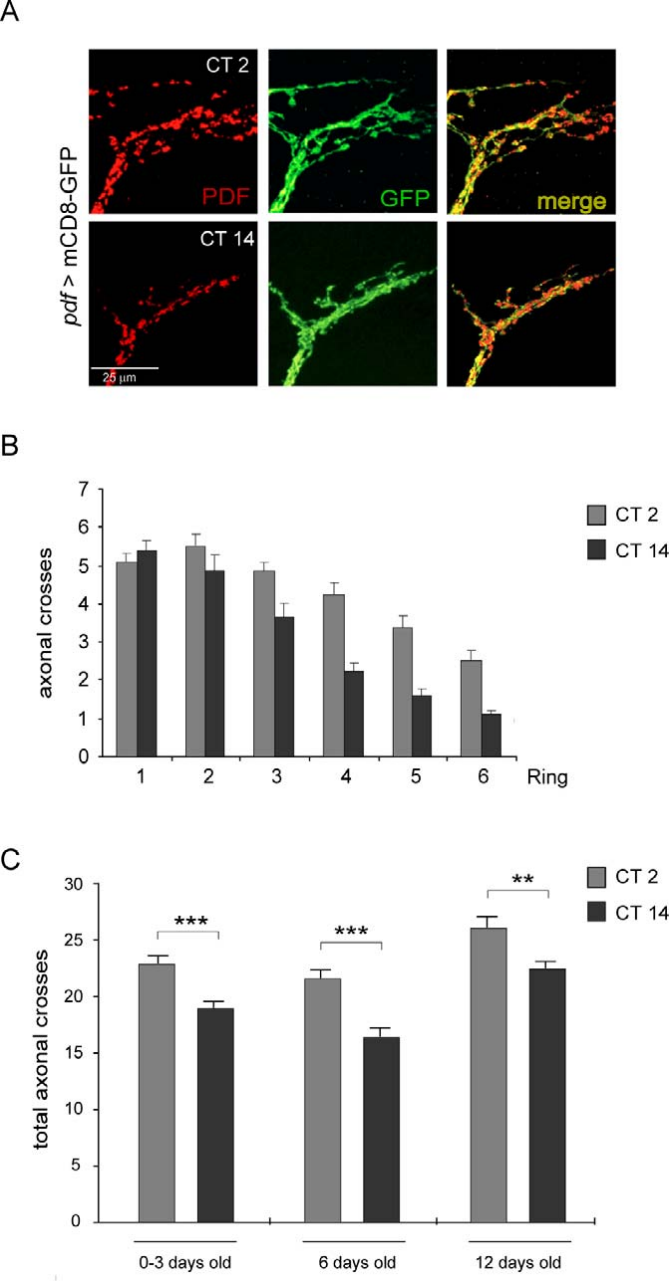
Changes in the Degree of Arborization of the PDF Circuit Are Preserved Under Constant Conditions (A–C) *pdf* >mCD8-GFP wild-type brains were dissected at CT2 and CT14 during the second day in constant darkness (DD2). CT refers to the time that has passed from the last lights OFF/ON transition. CT2 and CT14 indicate subjective day and night, respectively. (A) Confocal images representative of the subjective day (upper) and subjective night (bottom panels) stained with anti-PDF (red) and anti-GFP (green) antibodies. (B) The number of intersections is consistently lower during subjective night, although the PDF circuit is not particularly shortened. (C) Total axonal crosses are significantly different during subjective day and night, and the difference persists in older flies. Immunohistochemistry was performed at least three times for each group. Each timepoint represents a minimum of 60 brains; quantitation was performed blind. *** represents *p* < 0.0001 and ** represents *p* < 0.0005, non parametric Mann-Whitney test.

Daily changes in axonal morphology could merely be the direct response to a changing environment, as opposed to be regulated by an endogenous mechanism. To determine whether this structural plasticity was under the control of the endogenous biological clock whole mount adult brains from flies kept under constant darkness (DD) were fixed and dissected at circadian time (CT) 2 and 14 (Figure 2A) during the second day after transfer to constant conditions (DD2). Under these conditions, a significant decrease in the total number of axonal crosses in brains examined during subjective night was found (Figure 2C), when compared to those fixed during subjective day, consistent with a role of the circadian clock controlling axonal plasticity under both 12 h light/dark (LD) and DD conditions.

This decreased scoring for the nighttime configuration suggests a reduced overall complexity of the PDF axonal arbor at subjective night. This, in turn, could either result from a reduced number of higher order projections rendering a less branched arbor of similar length, or from projections with a decreased length, giving rise to an arbor of similar complexity but shorter branches (see a schematic representation of possible mechanisms in Figure S2B). To distinguish between these possibilities the number of projections representative of each segment (rings 1 through 6 in Figures 1D and S2A) were compared, and significant differences were observed along the entire region both in LD and DD, with the exception of the most proximal section (ring 1), supporting the notion that the reported remodeling is unlikely the result of circadian extension/retraction (Figures 1F and 2B).

Helfrich-Forster described how the PDF-associated signal along the midline slowly disappears right after eclosion [31]. Our experiments showed that not only did the neuropeptide signal decrease as the fly matured but also the GFP signal did, underscoring that this decrease most likely reflects a complete retraction of the PDF circuit itself (Figure S3). To discard a possible contribution of developmental changes to the cycling observed in circuit morphology at the dorsal protocerebrum 45 flies eclosed within a narrow 15 h-window kept under LD cycles were dissected at ZT2 and ZT14 during three consecutive days. Thus, flies dissected at ZT2 in the second or third day were older than those dissected the night before (at ZT14). When the analysis described in Figure 1D was performed, brains taken during the day displayed the characteristic open conformation, similar to that shown in Figure 1B, upper panel, thus precluding the possibility that the changes in morphology followed a developmental program (unpublished data).

To explore whether such circadian changes persisted into adulthood, six and 12 day-old flies were analyzed (Figure 2C). These timepoints were selected as they span a regular behavioral experiment. Interestingly, significant remodeling was observed even in older flies, reinforcing its relevance. No sexual dimorphism was found (Figures S4A–B), although a higher inter individual variability was observed in female flies.

### A Functional Clock Is Required for Circadian Structural Plasticity

**Figure 3:**
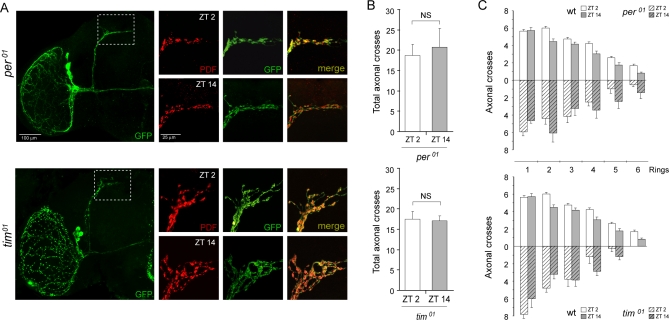
Structural Plasticity Is Under the Control of Clock Genes (A–C) The *pdf* >mCD8-GFP reporter was crossed into *per^01^* (top panel) and *tim^01^* (bottom panel) clockless mutants. Brains were dissected at ZT2 and ZT14 as explained in the legend to Figure 1. (A) Left: Low magnification view of a brain hemisphere in *per^01^* or *tim^01^* flies. Note the degree of defasciculation in *tim^01^* axon tract towards the dorsal protocerebrum. Right: Representative confocal images taken during the early day and early night, highlighting the differential effect of these mutations on the structure of the circuit. Immunohistochemistry was performed twice, each timepoint represents 15–21 brains; quantitation was performed blind. (B) No significant differences between the daytime and nighttime configuration were observed (*p* > 0.5, non parametric Mann-Whitney test). (C) *per^01^* flies display a less branched circuit of relatively normal length at all times; on the other hand, *tim^01^* flies display an over branching phenotype, characteristically shorter than wild-type brains.

Since this phenomenon was observed in the absence of direct effect of light on behavioral and molecular processes, we sought to determine the impact of the absence of a functional clock on the structure of the PDF circuit. Previous reports had described that both mutations affected the cycling in PDF intensity albeit to a different fashion [11]. *per^01^* mutants displayed constitutively low PDF immunoreactivity, meanwhile *tim^01^* mutants showed constantly high PDF signal. We introduced the *pdf* >mCD8-GFP transgenes into the *per^01^* and *tim^01^* mutant background and quantified the number of branches during early morning and night (ZT2 and ZT14, respectively); given the lack of a functional clock any differences would be attributed to the environmental condition. No significant difference between ZT2 and ZT14 was found in either mutant (Figures 3A–3C), further strengthening the involvement of the circadian clock in this process. The lack of rhythmicity could derive from different scenarios. Either the morphology of the dorsal projections in a mutant background would represent a given time point seen in wild-type brains (as interpreted for PDF staining [11]), or, alternatively, the PDF circuit could display a unique conformation never present in wild-type flies, suggesting these genes could be playing additional roles to those ascribed to core clock function. Consistent with our observations, Cantera and colleagues have recently reported that branching of a motor neuron is severely affected in the absence of these clock proteins, whereby in *per^01^* flies a reduced branching was observed, in stark contrast to the hyper branching characteristic of different *tim* null alleles [28].

Examples of structural plasticity have been reported along the years, such as the light-dependent changes in the axon terminals of bipolar cells in the rat retina [32] or the synaptic activity-dependent changes in the morphology of dendritic spines in cultured hippocampal neurons [33]. Recently, Pyza and colleagues reported daily changes in the properties of the L1 and L2 interneurons of the visual system of the house fly and in *Drosophila* [26,27]; interestingly, they showed a circadian oscillation in the size and spacing of the varicosities (alleged sites of peptide release) from the LNvs into the fly eye. These changes were proposed to convey circadian information out from the large LNvs [25].

Here we describe circadian changes in the structure of the circuit underlying rest-activity cycles in *Drosophila*. Such changes could result in differential activity within the circuit, fine-tuning the connection between the PDF circuit and its postsynaptic targets, or even reaching different targets along the day. Moreover, since PDF is responsible for synchronizing distinct clusters within the circadian network, this phenomenon could also impinge on the neuronal coupling between clock cells.

Although the underlying molecular mechanisms sustaining the circadian changes in structural plasticity are not understood, alternative scenarios are envisioned. Differences in the complexity of the axonal arbor could reveal a continuous neurite addition and retraction, reminiscent of that responsible of refining the connection between the retinal ganglion cell axons as they project into the optic tectum [34] which has been shown to be based on correlated activity [35]. Alternatively, this phenomenon could uncover a dynamic fasciculation/defasciculation process. In clear favor of such possibility is the observation that a number of cell adhesion molecules are under circadian regulation (as reported by microarray analysis [36,37]). The contribution of the cyclical accumulation and release of PDF containing vesicles to this phenomenon cannot be excluded.

Given the fundamental role of the establishment of the proper synaptic connections in any functional neuronal network, the ability of a certain neuron to reach its targets in a spatially and temporally regulated manner relies on a highly orchestrated set of processes. The hierarchical organization of the circadian network is one of the most thoroughly characterized in *Drosophila* [38]. The transmission of information from pacemaker cells, those that occupy the highest level within this hierarchy, is crucial for structuring the downstream rhythmic rest-activity cycles.

It has been shown that altering PDF expression in pacemaker cells or their electrical activity interferes with their proper output [39–41]. In this work we introduce a plastic change in the morphology of the PDF circuit as a candidate novel mechanism for transmitting clock information on a time-dependent manner: most likely, a circadian regulated variation in the establishment of synapses between pacemaker cells and their post synaptic targets.

## Materials and Methods

### Strains and fly rearing.

*y w* flies were used as wild-type controls. *pdf-gal*4 (chromosomes X and II) and UAS-mCD8 GFP lines (chromosomes II and III) were kindly provided by the Bloomington Stock Center. *tim^01^ and per^01^* were provided by Jeff Hall. The UAS-synaptobrevin-GFP line was generously provided by Bing Zhang. *Drosophila* cultures were maintained on a 12 h light/dark (LD) cycle on standard corn meal yeast agar medium at 25 °C in an environmental chamber.

### Immunohistochemistry.

Flies were entrained for 5 d in 12 h LD cycles at 25 °C before dissection. Most of the analysis was performed in young (0–3 d old) flies except for the experiments shown in Figure 2C, S3 and S4. Flies were decapitated either in LD or during the second day under constant darkness (DD2). Brains were fixed in 0.4% paraformaldehyde in PB (100mM KH_2_PO_4_,/Na_2_HPO_4_) and then rinsed three times in PT (PBS plus 0,1% Triton X-100). Brains were then blocked in 5% goat serum in PT for 2 h at room temperature (RT). After the blocking step tissue was incubated with primary antibodies, ON at 4 °C. The primary antibodies used were chicken anti-GFP (1/500, Upstate) and rabbit anti-PDF (1/1500, custom-made by NeoMPS). The secondary antibodies used were Cy2-conjugated donkey anti-chicken, Cy3- conjugated anti-rabbit (Jacksons Immunoresearch) diluted to a final concentration of 1/250, incubated for 2 h at RT. After staining, brains were washed 3 × 40 min and ON in PT and mounted in 80% glycerol (in PT).

### HRP staining.

Brains were fixed and dissected as described in the previous section. After blocking, brains were incubated with the primary antibody ON at 4 °C and washed 3 × 30 min at RT. The primary antibody used was rabbit anti-GFP (1/500, Molecular Probes). Brains were then incubated with the secondary antibody (1/250, Biotin-SP-conjugated Affinipure Donkey anti-rabbit IgG, Jackson Immunoresearch) for 2 h at RT and washed 3 × 15 min in PT. Then, they were incubated for 2 h at RT in AB (Vector laboratories) and after this step in DAB (0.3 ml H_2_O + 3.6 ml ClNi + 12.5 ml DAB in 1 ml PBS) for 1.5 min. The reaction was stopped with PB 0.1 M. Brains were washed in PBS 5 × 15 min and mounted in 80% glycerol (in PT).

### Confocal microscopy.

To visualize axon projections of small LNvs in *pdf*-*gal*4/UAS-mCD8-GFP, *pdf*-*gal*4/UAS-syb-GFP, *per^01^*; *pdf*-*gal*4/UAS-mCD8-GFP, and *pdf-gal4; tim^01^;* UAS-mCD8-GFP optical sections of whole brains were imaged on a Zeiss LSM510 confocal microscope. Images were taken with a 40× objective (water immersion) with an optical zoom of two. Galleries between seven and 19 images were projected in the *x*–*y* axis to obtain a reconstruction of the full trajectory of those axons.

### Quantitation of the axonal branching.

To quantify the axonal arbor at the dorsal protocerebrum an adaptation of the Sholl method [30] was used. Only the GFP associated signal was taken into account for the analysis. Six evenly spaced (10 μm) concentric rings centered at the point where the first dorsal ramification opens up were drawn on each brain hemisphere. The number of intersections of each projection with a particular ring (as illustrated in Figure 1D and Figure S2A) were counted. The number of intersections per ring as well as the total number of intersections were compared using non parametric statistical methods. Scoring was performed blindly. The total circuit length and the number of higher order processes could not be measured since discrete projections in the closed conformation were difficult to discern.

## Supporting Information

Figure S1 Axonal Arborizations of Small LNvs Extend Mostly in the Latero-Medial and Dorso-Ventral Axis 3-D reconstructions were analyzed to rule out the possible contribution of significant branching in the antero-posterior direction. All of the images included throughout the manuscript are contained within the *x*–*y* projections (*x*-axis, latero-medial; *y*-axis, dorso-ventral; *z*-axis, antero-posterior).
(A) Representative image of an antero-posterior view (right panel) of one of the brains whose axonal arborization was analyzed in the *x*–*y* projection (left panel). The lateral horn in the *x–y* projection corresponds to the anterior branch in the *y–z* projection (white arrowhead). The remaining axons extend fasciculated in the lateral to medial direction, and correspond to the posterior branch in the *y–z* projection (grey arrowhead). None of the lower order branches seen in the *x–y* image are distinguishable in the *y–z* reconstruction. The average depth of an axonal arbor in the antero-posterior direction is ∼7 mm.
(B) Brains from 6-d-old male and female flies were analyzed in DD2. The total axonal branching in the standard *x–y* view is shown in Figure S4. For each image, the total depth of the axonal arbor was quantified using the software provided by the confocal microscope, revealing no significant differences between timepoints for either male or female brains (*p* > 0.05, Mann Whitney test for non parametric samples).
(4.2 MB TIF)

Figure S2 Quantitation of the Daily Reorganization in the PDF Terminals at the Dorsal Protocerebrum (A) Representative confocal images of *pdf*>mCD8-GFP brains dissected at circadian time CT2 and CT14 in DD2. Six evenly spaced (10 μm) concentric rings centered at the point where the first dorsal ramification opens up were drawn on each brain hemisphere. The number of intersections per ring for each projection was counted and marked with white dots. The total number of crosses (*n*) is included in the upper corner. The method was adapted from Sholl [27].
(B) Alternative scenarios accounting for the structural plasticity observed in the dorsal protocerebrum. The differential complexity in the axonal arborizations of the PDF circuit during the day and night, reflected in fewer intersections in the latter, could derive from either higher order branches growing during the early day and disappearing later at night (additional branches), the lengthening and shortening of the more distal projections (retraction), or by controlling the degree of fasciculation of the major axonal processes (fasciculation), making it impossible to distinguish individual neurites at night (and thus retrieving a lower count). Although a clear picture has yet to emerge, we favor the notion of clock-controlled fasciculation (see text).
(2.8 MB TIF)

Figure S3 The PDF Signal along the Midline Disappears with Aging Brains from newly eclosed (less than 1 d old, upper panels) and 3–4 d old (lower panels) flies were dissected at ZT2 after five days of LD cycles and incubated with anti-PDF and anti-GFP antisera. Pupae and recently eclosed flies displayed an intense PDF signal along the midline and the area surrounding the sub oesophageal ganglion [31], which correlated well with GFP (top panels). The disappearance of both signals as flies aged (bottom panels) reveals that the absence of PDF signal in older flies could derive from pruning of these axons. Intermediate signals were also observed, where the sub oesophageal ganglion was still stained while the most distal neurites reaching the pars intercerebralis were missing (unpublished data).
(7.3 MB TIF)

Figure S4 Structural Plasticity Persists in Older Male and Female Flies Left panel: Representative confocal images of *pdf*>mCD8-GFP male and female brains, from six day-old (A) or 12 day-old (B) flies, are shown. Flies were fixed and dissected at CT2 and CT14 in DD2. Right panel: The quantitation of the total number of intersections between the concentric rings and the axonal projections is presented. Six day-old flies showed significant differences in the total number of axonal crosses in both males (*p* = 0.0233) and females (*p* < 0.0001). In contrast, 12 day-old males displayed a significantly different circuit complexity (*p* = 0.0017) while the females did not (*p* = 0.0717), albeit the nighttime conformation still showed a lower complexity. *, ** and *** refer to *p* < 0.05, *p* < 0.005, and *p* < 0.0001, respectively. Experiments were repeated at least three times with similar results.
(7.2 MB TIF)

## Abbreviations

CT: circadian time
DD: constant darkness
GFP: green fluorescent protein
LD: 12 h light/dark cycle
LNv: ventral lateral neuron
PDF: pigment dispersing factor
RT: room temperature
ZT: zeitgeber time
