# Supplementary figures and images for: Circadian Remodeling of Neuronal Circuits Involved in Rhythmic Behavior
Source: PLoS Biol. 2008 Mar 25;6(3):e69. doi: 10.1371/journal.pbio.0060069 (PMC2270325; doi:10.1371/journal.pbio.0060069)

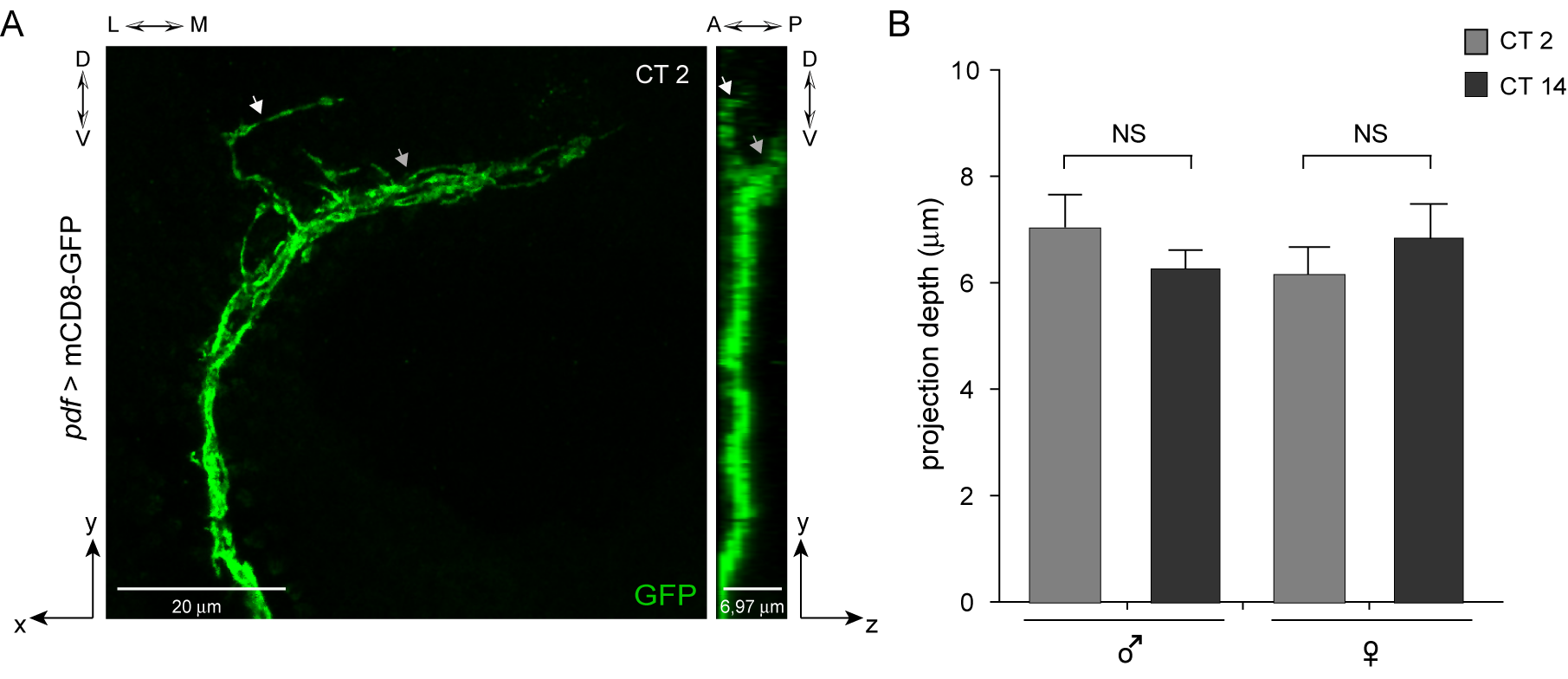

Supplement: Figure S1 — 3-D reconstructions were analyzed to rule out the possible contribution of significant branching in the antero-posterior direction. All of the images included throughout the manuscript are contained within the x–y projections (x-axis, latero-medial; y-axis, dorso-ventral; z-axis, antero-posterior). (A) Representative image of an antero-posterior view (right panel) of one of the brains whose axonal arborization was analyzed in the x–y projection (left panel). The lateral horn in the x–y projection corresponds to the anterior branch in the y–z projection (white arrowhead). The remaining axons extend fasciculated in the lateral to medial direction, and correspond to the posterior branch in the y–z projection (grey arrowhead). None of the lower order branches seen in the x–y image are distinguishable in the y–z reconstruction. The average depth of an axonal arbor in the antero-posterior direction is ∼7 mm. (B) Brains from 6-d-old male and female flies were analyzed in DD2. The total axonal branching in the standard x–y view is shown in Figure S4. For each image, the total depth of the axonal arbor was quantified using the software provided by the confocal microscope, revealing no significant differences between timepoints for either male or female brains (p > 0.05, Mann Whitney test for non parametric samples). (4.2 MB TIF) [file pbio.0060069.sg001.tif]

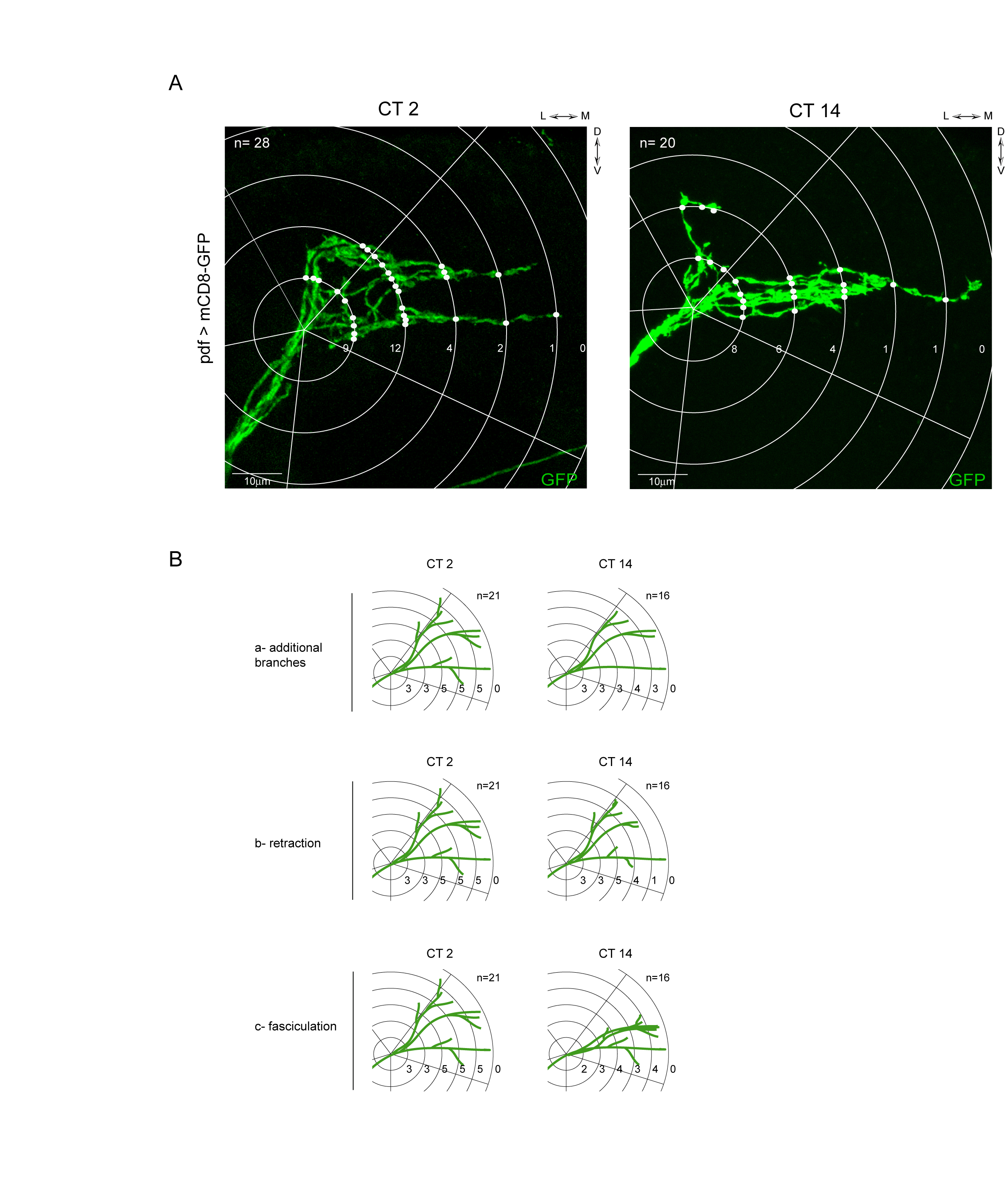

Supplement: Figure S2 — (A) Representative confocal images of pdf>mCD8-GFP brains dissected at circadian time CT2 and CT14 in DD2. Six evenly spaced (10 μm) concentric rings centered at the point where the first dorsal ramification opens up were drawn on each brain hemisphere. The number of intersections per ring for each projection was counted and marked with white dots. The total number of crosses (n) is included in the upper corner. The method was adapted from Sholl [27]. (B) Alternative scenarios accounting for the structural plasticity observed in the dorsal protocerebrum. The differential complexity in the axonal arborizations of the PDF circuit during the day and night, reflected in fewer intersections in the latter, could derive from either higher order branches growing during the early day and disappearing later at night (additional branches), the lengthening and shortening of the more distal projections (retraction), or by controlling the degree of fasciculation of the major axonal processes (fasciculation), making it impossible to distinguish individual neurites at night (and thus retrieving a lower count). Although a clear picture has yet to emerge, we favor the notion of clock-controlled fasciculation (see text). (2.8 MB TIF) [file pbio.0060069.sg002.tif]

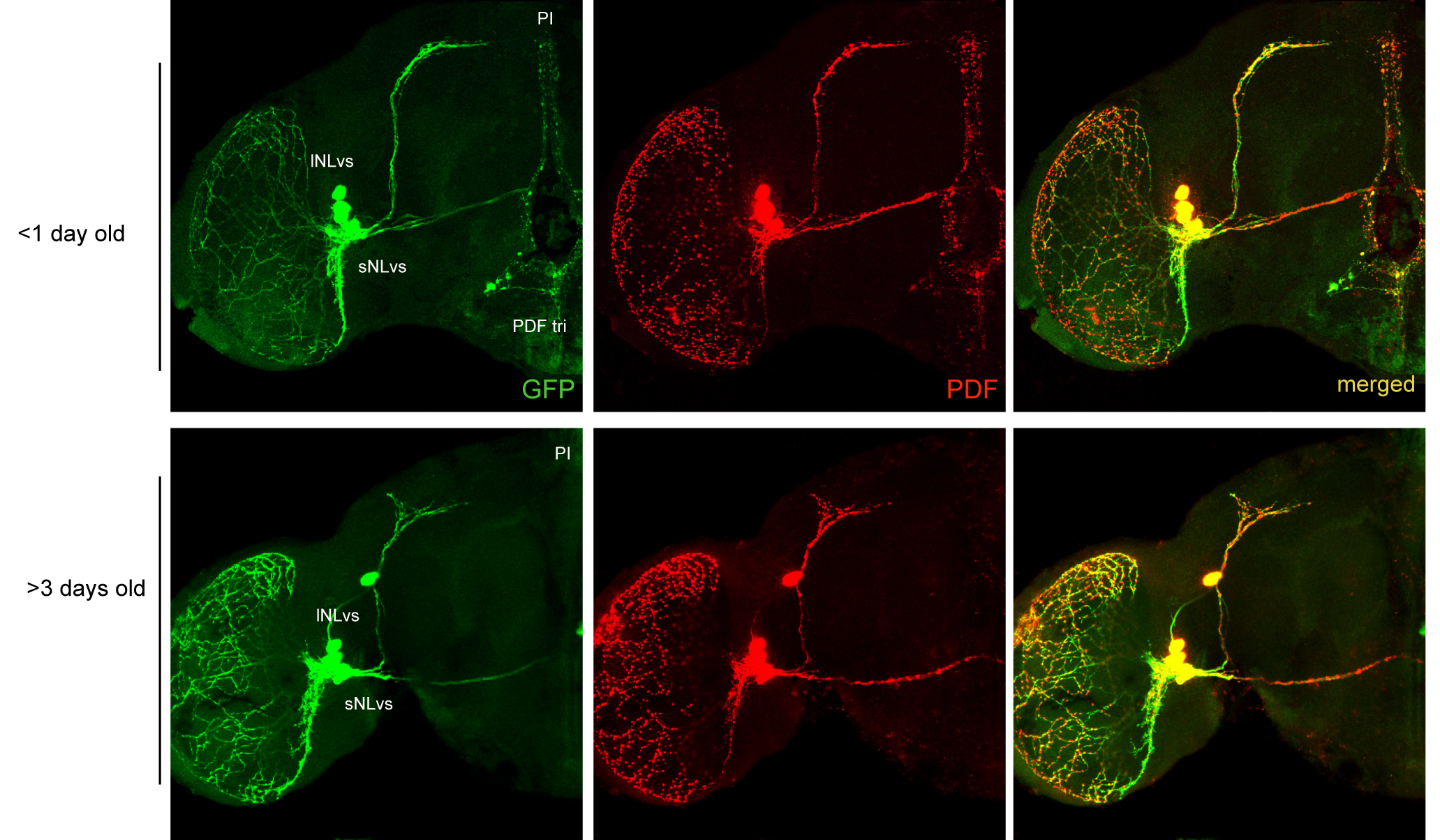

Supplement: Figure S3 — Brains from newly eclosed (less than 1 d old, upper panels) and 3–4 d old (lower panels) flies were dissected at ZT2 after five days of LD cycles and incubated with anti-PDF and anti-GFP antisera. Pupae and recently eclosed flies displayed an intense PDF signal along the midline and the area surrounding the sub oesophageal ganglion [31], which correlated well with GFP (top panels). The disappearance of both signals as flies aged (bottom panels) reveals that the absence of PDF signal in older flies could derive from pruning of these axons. Intermediate signals were also observed, where the sub oesophageal ganglion was still stained while the most distal neurites reaching the pars intercerebralis were missing (unpublished data). (7.3 MB TIF) [file pbio.0060069.sg003.tif]

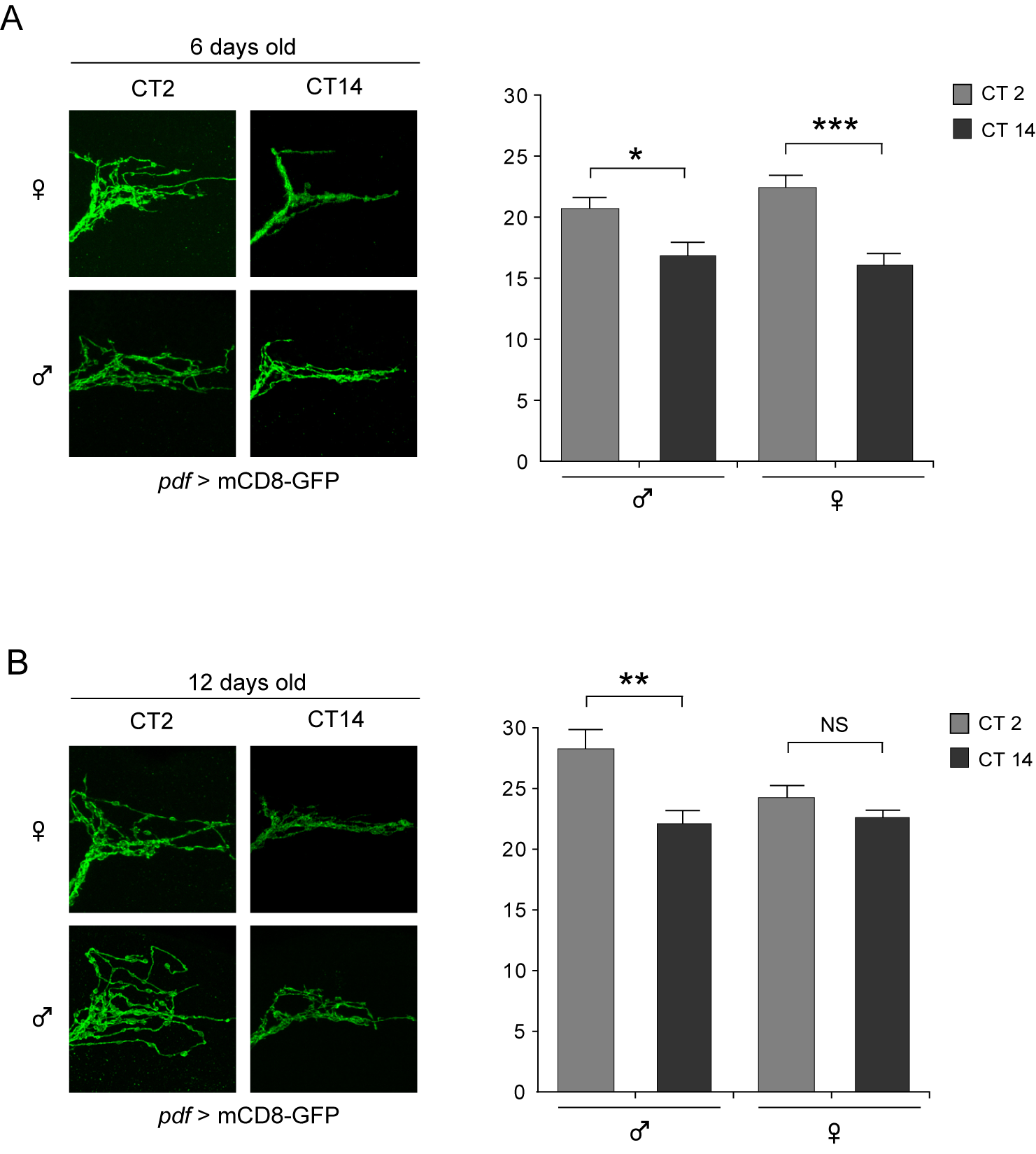

Supplement: Figure S4 — Left panel: Representative confocal images of pdf>mCD8-GFP male and female brains, from six day-old (A) or 12 day-old (B) flies, are shown. Flies were fixed and dissected at CT2 and CT14 in DD2. Right panel: The quantitation of the total number of intersections between the concentric rings and the axonal projections is presented. Six day-old flies showed significant differences in the total number of axonal crosses in both males (p = 0.0233) and females (p < 0.0001). In contrast, 12 day-old males displayed a significantly different circuit complexity (p = 0.0017) while the females did not (p = 0.0717), albeit the nighttime conformation still showed a lower complexity. *, ** and *** refer to p < 0.05, p < 0.005, and p < 0.0001, respectively. Experiments were repeated at least three times with similar results. (7.2 MB TIF) [file pbio.0060069.sg004.tif]
